# Comprehensive transcriptomic analysis of three varieties with different brown planthopper-resistance identifies leaf sheath lncRNAs in rice

**DOI:** 10.1186/s12870-023-04374-w

**Published:** 2023-07-22

**Authors:** Kai Liu, Xiaozhi Ma, Luyao Zhao, Xiaofeng Lai, Jie Chen, Xingxuan Lang, Qunxin Han, Xiaorong Wan, Chunmei Li

**Affiliations:** 1 Guangzhou Key Laboratory for Research and Development of Crop Germplasm Resources, Guangdong University Key Laboratory for Sustainable Control of Fruit and Vegetable Diseases and Pests & Key Laboratory of Green Prevention and Control on Fruits and Vegetables in South China, Ministry of Agriculture and Rural Affairs, Zhongkai University of Agriculture and Engineering, Guangzhou, 510225 China; 2 Guangdong Provincial Key Laboratory of New Technology in Rice Breeding, Rice Research Institute, Guangdong Academy of Agricultural Sciences, Guangzhou, 510642 China

**Keywords:** Resistant rice, *Nilaparvata lugens*, Transcriptome, Long non-coding RNAs, Expression analysis

## Abstract

**Background:**

Long non-coding RNAs (lncRNAs) have been brought great attention for their crucial roles in diverse biological processes. However, systematic identification of lncRNAs associated with specialized rice pest, brown planthopper (BPH), defense in rice remains unexplored.

**Results:**

In this study, a genome-wide high throughput sequencing analysis was performed using leaf sheaths of susceptible rice Taichung Native 1 (TN1) and resistant rice IR36 and R476 with and without BPH feeding. A total of 2283 lncRNAs were identified, of which 649 lncRNAs were differentially expressed. During BPH infestation, 84 (120 in total), 52 (70 in total) and 63 (94 in total) of differentially expressed lncRNAs were found only in TN1, IR36 and R476, respectively. Through analyzing their cis-, trans-, and target mimic-activities, not only the lncRNAs targeting resistance genes (NBS-LRR and RLKs) and transcription factors, but also the lncRNAs acting as the targets of the well-studied stress-related miRNAs (miR2118, miR528, and miR1320) in each variety were identified. Before the BPH feeding, 238 and 312 lncRNAs were found to be differentially expressed in TN1 vs. IR36 and TN1 vs. R476, respectively. Among their putative targets, the plant-pathogen interaction pathway was significantly enriched. It is speculated that the resistant rice was in a priming state by the regulation of lncRNAs. Furthermore, the lncRNAs extensively involved in response to BPH feeding were identified by Weighted Gene Co-expression Network Analysis (WGCNA), and the possible regulation networks of the key lncRNAs were constructed. These lncRNAs regulate different pathways that contribute to the basal defense and specific resistance of rice to the BPH.

**Conclusion:**

In summary, we identified the specific lncRNAs targeting the well-studied stress-related miRNAs, resistance genes, and transcription factors in each variety during BPH infestation. Additionally, the possible regulating network of the lncRNAs extensively responding to BPH feeding revealed by WGCNA were constructed. These findings will provide further understanding of the regulatory roles of lncRNAs in BPH defense, and lay a foundation for functional research on the candidate lncRNAs.

**Supplementary Information:**

The online version contains supplementary material available at 10.1186/s12870-023-04374-w.

## Background

The brown planthopper (BPH) (*Nilaparvata lugens* Stål) is the most widespread and devastating pest of rice (*Oryza sativa*), sucking phloem of sheath, and thus causing direct damage and transmitting viral diseases to rice plants [1, 2]. Rice directly encounters BPH’s attacks via endogenous genes [3–7], secondary metabolites such as flavonoids [8, 9], and signaling networks in which phytohormones have central roles, particularly salicylic acid (SA) and jasmonic acid (JA) [7, 10–13]. Maintenance of cell wall integrity and lignification acts as physical barriers [7]. BPH defense of rice is also indirectly regulated via exogenous chemicals, honeydew-associated elicitor, silicon and biochar amendments, and salivary protein of BPH as elicitor or effector [14].

To date, about 40 BPH resistance genes have been detected in cultivated and wild species of rice through forward genetics [2]. Within these isolated genes, *Bph14* [10, 15] and *Bph1*/*2*/*7*/*9*/*10*/*18*/*21*/*26* [11] encode NBS-LRR proteins, whereas *Bph3* encode lectin receptor-like receptors [16]. *Bph14* was further demonstrated to interact with transcription factors OsWRKY46 and OsWRKY72, thereby enhancing the expression of the receptor-like cytoplasmic kinase gene *RLCK281* and the callose synthase gene and resulting in BPH resistance [15]. Besides, reverse genetics approach identified OsGID1 (the gibberellin (GA) receptor) and OsSLR1 (a negative regulator of GA pathway) to enhance BPH defense through elevating lignin levels [16, 17]. R2R3 MYB transcription factor confers BPH resistance by upregulating *OsPALs* (phenylalanine ammonia lyase) in phenylpropanoid pathway to increase accumulation of SA and lignin [7]. Recent studies also revealed non-coding RNA, such as microRNAs, responding to BPH feeding [18, 19]. Both OsmiR396 and OsmiR156 have been shown to negatively regulate BPH resistance in rice by affecting the flavonoid and JA biosynthesis, respectively [8, 20]. However, information related to long non-coding RNAs (lncRNAs) involved in defense against BPH feeding remains unexplored.

LncRNAs are a group of non-coding RNAs more than 200nt, classified into intergenic lncRNA (lincRNA), intronic lncRNA (incRNA), natural antisense lncRNA (NAT), and sense lncRNA based on their origins on the chromosomes [21, 22]. Plant lncRNAs function widely in growth, development, reproduction, and resistance to abiotic and biotic stresses in various species [23]. Most functionally well-characterized lncRNAs such as *COOLAIR* (cold induced long antisense intragenic RNA), *COLDAIR* (COLD ASSISTED INTRONIC NONCODING RNA), *COLDWRAP* (Cold of Winter-induced noncoding RNA from the Promoter), *MAS* (natural antisense of MADS AFFECTING FLOWERING 4) and *FLORE* (natural antisense of *CDF5*) in *Arabidopsis*, *Ef-cd* (Early flowering-completely dominant) in rice and *VAS* (*TaVRN1* alternative splicing) in wheat (*Triticum aestivum*), were involved in flowering [24–32]. Nevertheless, in rice, only seven lncRNAs have been demonstrated to be engaged in yield (*LAIR*), flowering (*Ef-cd*), fertility (*LDMAR*, *MISSEN*, and *PMS1T*), leaf development (*TL*), and bacterial blight resistance (*ALEX1*) [31, 33–40]. However, the way how lncRNAs act in plants defending insects remained largely unknown until lncRNAs responding to *Manduca sexta* attack were identified in wild tobacco (*Nicotiana attenuata*). Subsequently, silencing *JAL1* and *JAL3*, two early responding lincRNAs, showed decreased JA-mediated herbivore defense in wild tobacco [41].

To better understand the rice defense against BPH feeding and characterize defense-related lncRNAs, we analyzed the transcriptomic profile of mRNAs and lncRNAs in two resistant (IR36 and R476) and one susceptible (TN1) indica rice varieties treated with and without BPH feeding for 24 h. Subsequently, BPH defense-related lncRNAs were systematically identified and characterized. Integrating the analysis on cis-, trans-, and target mimic-activities, the putative regulatory function of these lncRNAs to mRNAs were predicted and analyzed. These results provided new insights into the regulatory mechanism of lncRNAs in rice during BPH infestation, and laid a foundation for functional research on the candidate BPH defense-related lncRNAs.

## Results

### Evaluation of BPH resistance

**Fig. 1:**
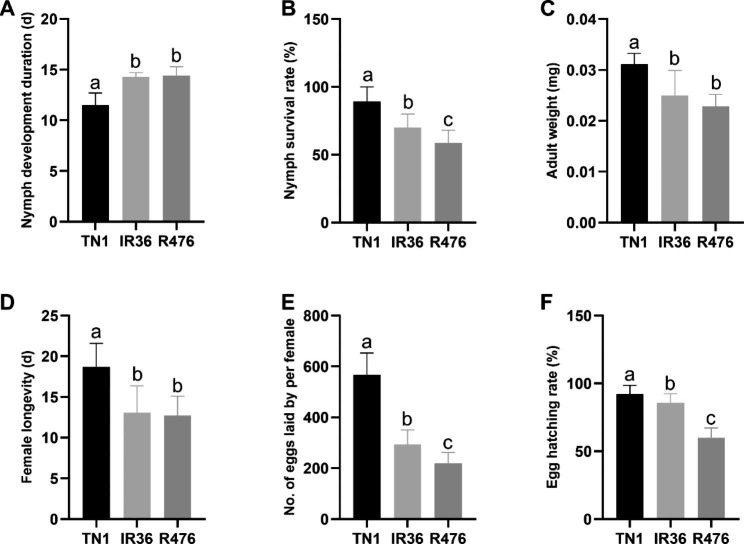
The ecological fitness of the brown planthopper (BPH) that fed on TN1, IR36 and R476. **(a)** Nymph development duration. **(b)** Nymph survival rate. **(c)** Adult weight. **(d)** Female longevity. **(e)** No. of eggs laid by per female. **(f)** Egg hatching rate. Each index of ecological fitness of the BPH were repeated at least for 12 times. The values are presented as the mean ± SEM. Values with different letters are statistically different by the Ducans’ multiple range test (*P* < 0.05)

To evaluate the BPH resistance of each variety, we analyzed the ecological fitness of BPH reared on TN1, IR36 and R476. Compared to feeding on TN1, feeding on IR36 and R476 resistant rice varieties significantly prolonged the nymph development duration from 11.49 to 24.19% and 25.24%, and decreased the nymph survival rate from 89.35 to 70.01% and 58.85% (Fig. 1a, b). The lower adult weight of BPH (TN1: 3.11 mg/female, IR36: 2.49 mg/female, R476: 2.28 mg/female) reared on IR36 and R476 variety indicated that IR36 and R476 was not suitable for BPH nymph growth, as did the decreased female longevity from 18.72 d to 13.07 d and 12.72 d (Fig. 1c, d). There were substantial differences in fecundity among the three groups, BPH reared on TN1 laid 567.39 eggs per female, which was significantly greater than that of BPH fed on IR36 and R476, which produced 293.60 and 219.06 eggs per female (Fig. 1e), and BPH fed on R476 rice varieties produced the lowest number of eggs. In addition, feeding on IR36 and R476 significantly shorten the BPH egg hatching rate from 92.35 to 85.83% and 59.81% (Fig. 1f). Furthermore, R476 reduced the nymph survival rate and fecundity of BPH obviously more than IR36 (Fig. 1b, e, f). Taken together, these results confirmed the BPH resistance of IR36 and R476 and suggested that R476 had a more resistance phenotype than IR36.

### Gene expression profile of susceptible and resistant rice after BPH feeding

To investigate the mRNA and lncRNA expression profiles in response to BPH infestation, transcriptome sequencing of BPH-susceptible (TN1) and resistant rice (IR36 and R476) at 24 h after BPH infestation was performed. Eighteen samples (three biological replicates for each) were collected for sequencing and analysis. We obtained more than 100 million clean reads from each sample. More than 90% of the clean reads from samples without BPH feeding were mapped onto the R498 reference genome (Supplementary Table S1). Approximately 70–90% of the clean reads in these samples were mapped to a unique gene (Supplementary Table S1). However, the mapping rate of the clean reads from samples after BPH feeding were relatively lower, ranging from 28.94 to 54.16% (Supplementary Table S1). Finally, 3071 (2086 upregulated and 985 downregulated), 2389 (1574 upregulated and 815 downregulated), and 2323 (1748 upregulated and 575 downregulated) differentially expressed mRNA (DEGs) were detected in TN1, IR36, and R476 after BPH infestation, respectively (Supplementary Fig. S1a). Venn diagram showed that there were 1259 common DEGs in all the three varieties (Supplementary Fig. S1b), 1045 (Supplementary Fig. S1c) and 212 (Supplementary Fig. S1d) of which were upregulated and downregulated, respectively, while the rest 2 of which showed differed expression in the three varieties after BPH feeding (Supplementary Fig. S1b, c, d, e, f, g). GO (Gene Ontology) analysis was conducted to understand the function of the DEGs. The top 20 enriched GO of biological processes showed that several GO terms related to defense were significantly enriched in all the 3 varieties, such as carbohydrate metabolic process, response to wounding, response to oxidative stress and regulation of jasmonic acid meditated signaling pathway (Supplementary Fig. S2a, b, c). The top 20 enriched Kyoto Encyclopedia of Genes and Genomes (KEGG) [42–44] pathways included pathways such as phenylpropanoid biosynthesis and flavonoid biosynthesis (Supplementary Fig. S2d, e, f), which have been suggested to play different roles in Nipponbare and C331 during BPH infestation [45]. These results indicated their necessary roles in BPH defense.

### Identification and characterization of lncRNAs in rice

**Fig. 2:**
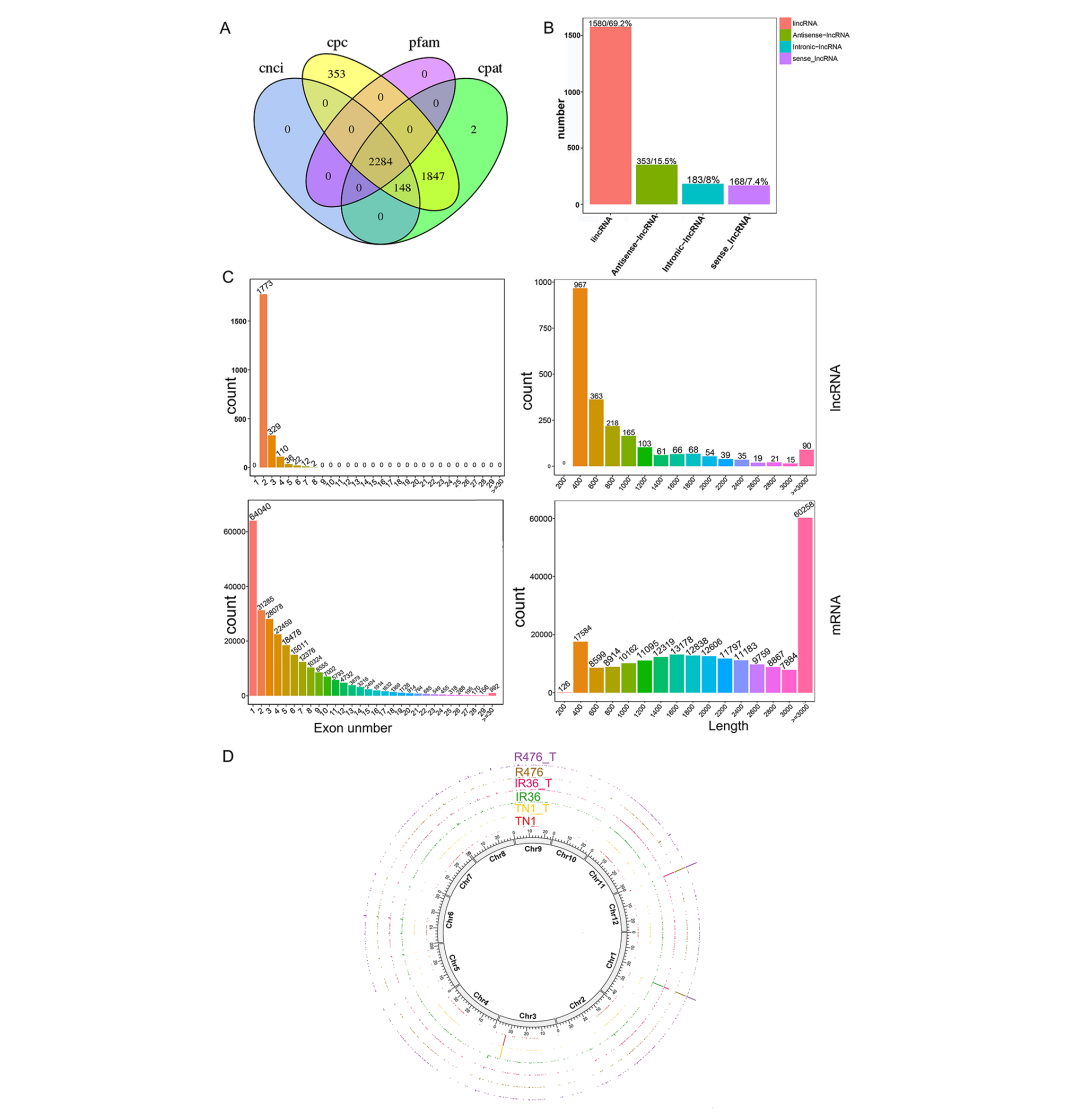
Identification and characterization of lncRNAs in TN1, IR36 and R476. **(a)** Venn diagram analysis of lncRNAs using CNCI, CPC, Pfam, and CPAT software. **(b)** Propotion of different kinds of lncRNAs. **(c)** Comparison of the exon number and trancript length between the identified lncRNAs and mRNAs. **(d)** Expression levels and genomic locations along the chromesomes of all the lncRNAs in all samples displayed by Circos program

From 18 samples, a total of 2283 lncRNAs were identified based on their read coverage, transcript length and protein coding potential revealed by Coding Potential Calculator (CPC), Coding-Non-Coding Index (CNCI), Pfam and Coding Potential Assessment Tool (CPAT) (Fig. 2a). Amongst these identified lncRNAs, the most abundant class was lincRNA (1597/69.2%), followed by antisense-lncRNA (353/15.5%), and the least abundant was sense lncRNA (168/7.4%) (Fig. 2b). All the identified lncRNAs and their classified types are shown in Supplementary Table S2. The exon number and length of the lncRNAs were significantly lower than those of mRNAs and few lncRNAs contained more than four exons (Fig. 2c). By using the Circos program [46], an expression distribution of lncRNAs from TN1, IR36, and R476 with and without BPH feeding along the 12 chromosomes of rice (Chr1–Chr12) was constructed (Fig. 2d). These lncRNAs exhibited no obvious preference for any particular genomic location (Fig. 2d). The expression levels of all the identified lncRNAs are listed in Supplementary Table S2. Six lncRNAs and six protein-coding genes were selected for validation with RT-qPCR. Gene expression trends in RT-qPCR is in general agreement with gene expression trends from RNA-sequencing data (Supplementary Fig. S3).

### Identification of lncRNAs related to BPH resistance

To identify lncRNAs responding to BPH, a total of 120, 70 and 94 differentially expressed lncRNAs (DELs) were found individually in TN1, IR36 and R476 after 24 h of BPH feeding. The resistant varieties IR36 and R476 encompassed less DELs than the susceptible variety TN1. However, most of the DELs in TN1 were downregulated, whereas the resistant varieties had more upregulated lncRNAs (Supplementary Fig. S4a). Additionally, venn diagram showed that 84, 52 and 63 lncRNAs were specifically differentially expressed in TN1, IR36 and R476, respectively (Supplementary Fig. S4b). Interestingly, there were 7 overlapping DELs that were upregulated in all the 3 varieties after BPH feeding (Supplementary Fig. S4b, c, d). These results demonstrated that lncRNAs responded to BPH feeding in resistant and susceptible varieties, and the 7 overlapping DELs probably played a vital role in the BPH response.

To determine whether the expression of lncRNAs in resistant rice was different from that in susceptible rice, the expression of lncRNAs in IR36 or R476 before BPH attack was first compared to TN1. It was found that there were 238 and 312 DELs in the TN1 vs. IR36 and TN1 vs. R476 comparisons, respectively (Supplementary Fig. S4a). The lncRNAs were differentially expressed between resistant and susceptible varieties before BPH feeding. Venn diagram showed that there were 32 upregulated (intersection of ‘TN1 vs IR36 up’ and ‘TN1_T vs IR36_T up’ in Supplementary Fig. S4f) and 54 downregulated (intersection of ‘TN1 vs IR36 down’ and ‘TN1_T vs IR36_T down’ in Supplementary Fig. 4e) lncRNAs in IR36 before and after BPH attack. Nevertheless, the remained DELs in IR36 before BPH attack showed no obvious difference after BPH attack (Supplementary Fig. S4e, f; Supplementary Table S2). Additionally, there were 109 common DELs in total in intersection of ‘TN1 vs. R476’ and ‘TN1_T vs. R476_T’ in Supplementary Fig. S4e, 50 of which were up regulated (intersection of ‘TN1 vs. R476 up’ and ‘TN1_T vs. R476_T up’ in Supplementary Fig. S4f) and 58 of which were down regulated (intersection of ‘TN1 vs. R476 down’ and ‘TN1_T vs. R476_T down’ in Supplementary Fig. S4g) before and after BPH attack in R476. However, the remained 1 lncRNA (MSTRG.32859.9) showed lower expression in R476 before BPH attack, while higher expression after BPH attack, compared to TN1 (Supplementary Fig. S4f, g; Supplementary Table S2). Particularly, MSTRG.32859.9 was induced after BPH attack specifically in R476, indicating that MSTRG.32859.9 might be involved in BPH defense response in R476.

### Identification of potential target genes of lncRNAs

LncRNAs regulate gene expression through cis and trans routes. Cis-acting lncRNAs regulate their neighboring genes colocalized on the same chromosome epigenetically and only at the transcriptional level, while trans-acting lncRNAs influence gene expression in diverse biological processes at the transcriptional or post-transcriptional level [47]. In this study, a total of 2168 lncRNAs were predicted to have cis-function, while 1391 lncRNAs were predicted to have trans-function, in which 1276 lncRNAs were predicted to act in both cis and trans regulatory ways (Supplementary Table S2). More than that, lncRNAs could function as miRNA targets to form a ceRNA network and thus regulate the expression of target transcripts of the same miRNAs [48]. Hence, we mined for lncRNAs that show complementarity with one of the 713 known rice miRNAs by psRNATarget (https://www.zhaolab.org/psRNATarget/). If such complementarity-based binding between lncRNA and miRNA was predicted, the lncRNA was predicted to have the target mimic activity [49]. However, much less than the number of predicted cis- or trans- functional lncRNAs, a total of 270 lncRNAs potentially function as target mimic of miRNA (Supplementary Table S2, S3). These results indicated that a single lncRNA might function by targeting different genes in multiple ways.

### Most of the DELs specifically responded to BPH feeding in different varieties

**Fig. 3:**
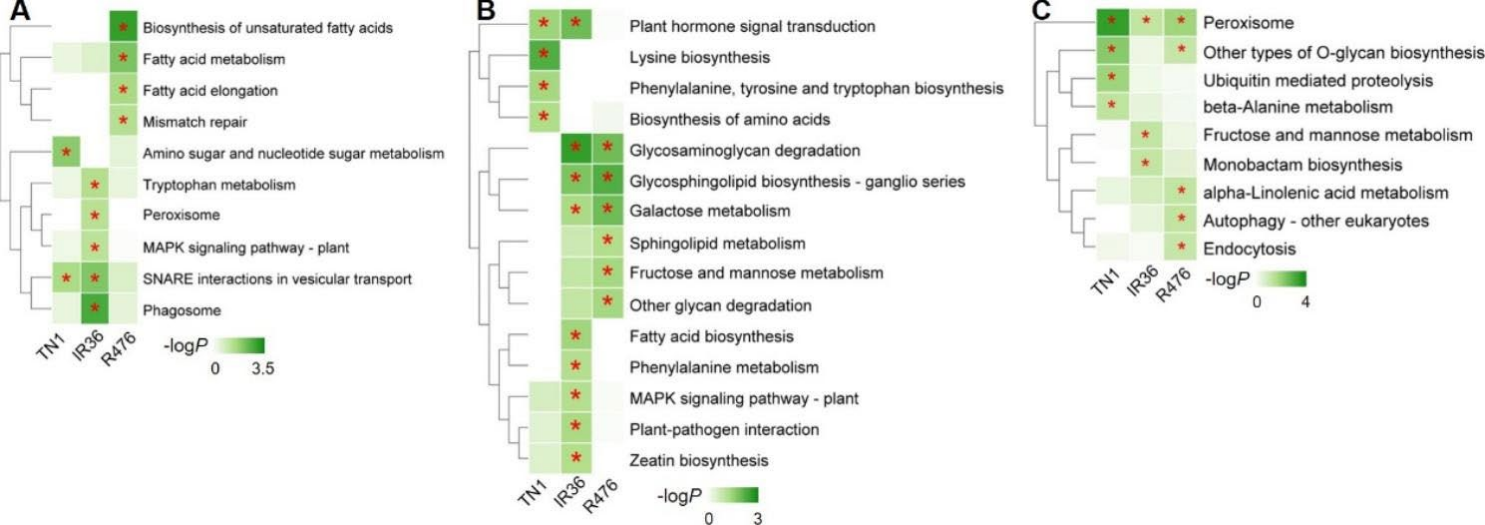
KEGG analysis of cis- **(a)**, trans- **(b)**, and target mimic-function **(c)** of DELs specifically response after BPH feeding in TN1, IR36 and R476. -log*P* was used for heatmap. * indicates *P* ≤ 0.05

As the venn diagram shows (Supplementary Fig. S4b), 84 (120 in total), 52 (70 in total) and 63 (94 in total) of DELs were found only in TN1, IR36 and R476 during BPH infestation, respectively, indicating that most of the DELs specifically responded to BPH feeding in different varieties and that the lncRNAs had less conservation than mRNAs. To understand the function of these lncRNAs, their cis-, trans-, and target mimic-activity was analyzed (Supplementary Table S4, S5, S6). Moreover, the GO and KEGG analysis of the putative targets were further conducted, the enriched GO terms in the biological processes and KEGG pathways (*P* ≤ 0.05) were listed in Supplementary Table S7. Intriguingly, KEGG analysis of the putative targets showed that less pathways were enriched in the susceptible variety TN1 than those in the resistant varieties IR36 and R476 (Fig. 3; Supplementary Table S7). The specific DELs in IR36 could function in signaling transduction pathways such as MAPK signaling pathway, plant hormone signal transduction and plant-pathogen interaction, and primary metabolisms such as galactose, whereas R476 possessed specific DELs involved in pathways mostly referring to many primary metabolisms such as fatty acid metabolism, and carbohydrate metabolism including galactose metabolism, fructose and mannose metabolism and so on (Fig. 3a, b, c). These results indicated the differential roles of lncRNAs against BPH in different varieties with different resistance genes.

**Fig. 4:**
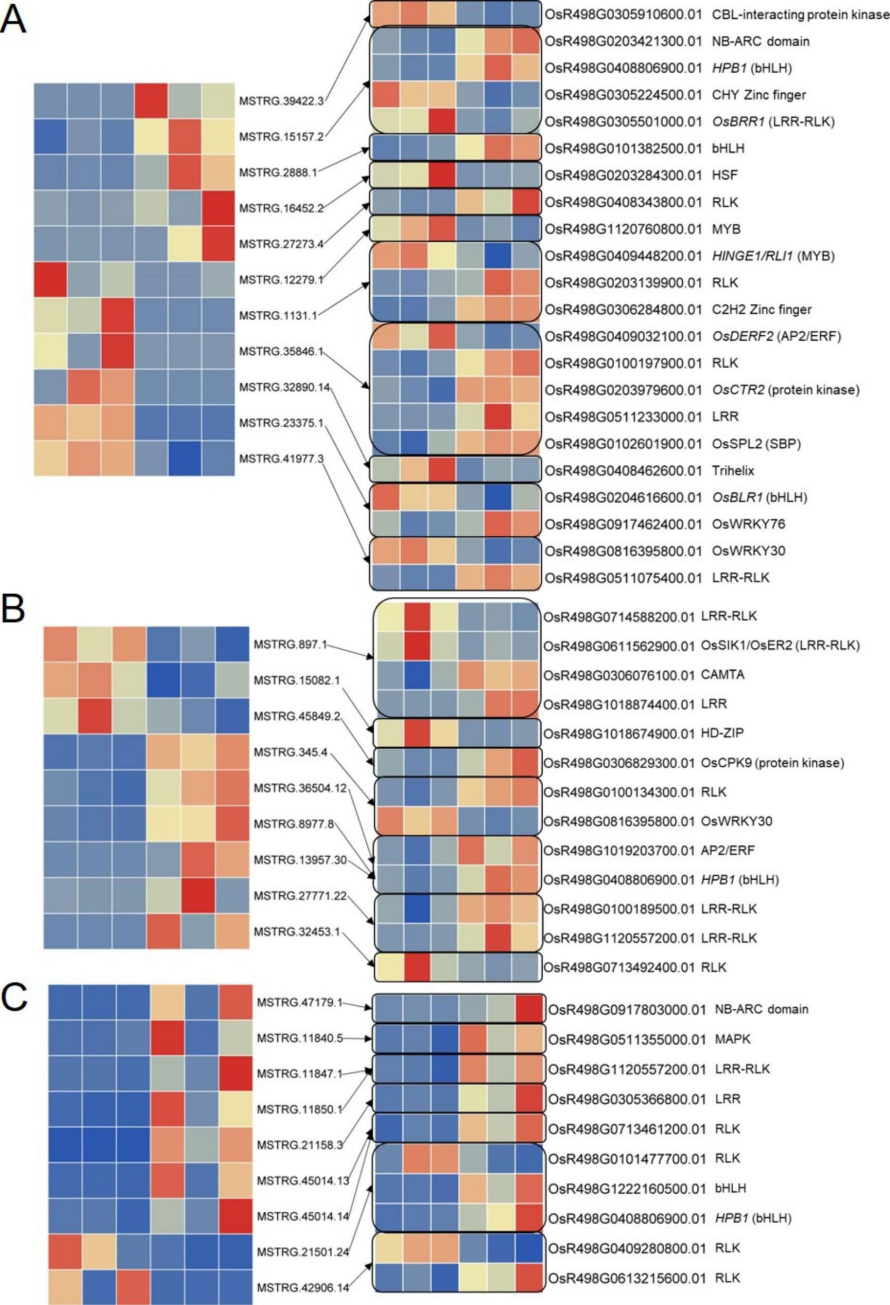
Diagram of DELs and their putative targets transcription factors, NBS-LRR and protein kinase in TN1 **(a)**, IR36 **(b)** and R476 **(c)**

The specific DELs, whose target genes were differentially expressed after BPH feeding, were further analyzed (Supplementary Table S8, S9, S10). These differentially expressed targets (DE targets) were submitted to the Plant Transcription Factor Database (http://planttfdb.gao-lab.org/prediction.php) [50] to identify transcription factors. Twelve transcription factors regulated by 9 DELs were identified in TN1 (Supplementary Table S8). Five transcription factors regulated by 6 DELs were annotated in the IR36 (Supplementary Table S9). However, in R476, only MSTRG.21501.24 putatively upregulate 2 bHLH transcription factors through target mimic activity (Supplementary Table S10). These results suggested that BPH elicited specific lncRNAs in susceptible variety TN1 might participate in defending BPH infestation through regulating more transcriptional factors than resistant varieties IR36 and R476. It was noteworthy that a single lncRNA was found to have more than one differentially expressed target transcription factors. For instance, OsR498G0306284800.01 (C2H2-type zinc finger) and OsR498G0409448200.01 (MYB-CC) regulated by the trans-activity of MSTRG1131.1. Similar case was found in MSTRG.35846.1 c by competing osa-miR529b with OsR498G0102601900.01 (SPL) and OsR498G0409032100.01 (ERF). Not only that, it was noticed that MSTRG.15157.2 in TN1, three lncRNAs (MSTRG.8977.8, MSTRG.36504.12, and MSTRG.13957.30) in IR36, and MSTRG.21501.24 in R476 were predicted to target the same miRNA osa-miR1439 to regulate its target bHLH (OsR498G0408806900.01) (Supplementary Table S8, S9, S10; Fig. 4), suggesting a vital role of osa-miR1439 in BPH defense in resistant rice and that different lncRNAs could target the same miRNA to regulate the target mRNA.

Some evidences indicate that plants’ defense to insects shares many similarities with their defense to pathogens [2, 51–53]. Most resistance proteins in plant contain a nucleotide-binding site including a P-loop, a C-terminal extension that forms a four-helix bundle (NBS, or NB-ARC domain), and a series of leucine-rich repeats (LRRs) domain, which are termed as NBS-LRR proteins. Within the known BPH resistant genes identified by forward genetics, most of them encode NBS-LRR resistant protein[2]. Beyond NBS-LRR family, receptor-like kinases/receptor-like protein (RLK/RLP) also contribute largely to plant defense [54]. Therefore, the DE targets potentially regulated by the specific DELs in each variety were also submitted to pfam (http://pfam.xfam.org/) to identify NBS-LRR proteins by analyzing NB-ARC (PF00931) and LRR (PF00560) domain, and were also subjected to iTAK (http://itak.feilab.net/cgi-bin/itak/index.cgi) [55] to identify protein kinases. Eventually, lncRNAs targeting differentially expressed NBS-LRR and protein kinase genes were found in all the three varieties (Supplementary Table S8, S9, S10; Fig. 4). These results demonstrated lncRNAs targeting resistant genes could be involved in defending against BPH feeding in either susceptible or resistant varieties.

### Common lncRNAs response to BPH feeding in susceptible and resistant varieties

It was found that there were 7 DELs commonly responding to BPH infestation in 3 varieties, which might be vital for the common downstream defense response to BPH. To further understand the roles of 7 DELs, their cis-, trans-, and target mimic-activity analysis was performed. Six DELs were found to have 13 DE targets after BPH feeding in all the 3 varieties (Supplementary Table S11), referring to proteins or pathways related to defense such as osmotin-like protein [56], CYP450 [57], flavonol synthase in flavonoid biosynthesis (ko00941) [9], RLK in plant-pathogen interaction (ko04626), lipoxygenase 8 in alpha-Linolenic acid metabolism (ko00592) [58], and beta-glucosidase in phenylpropanoid biosynthesis (ko00940) [45].

**Fig. 5:**
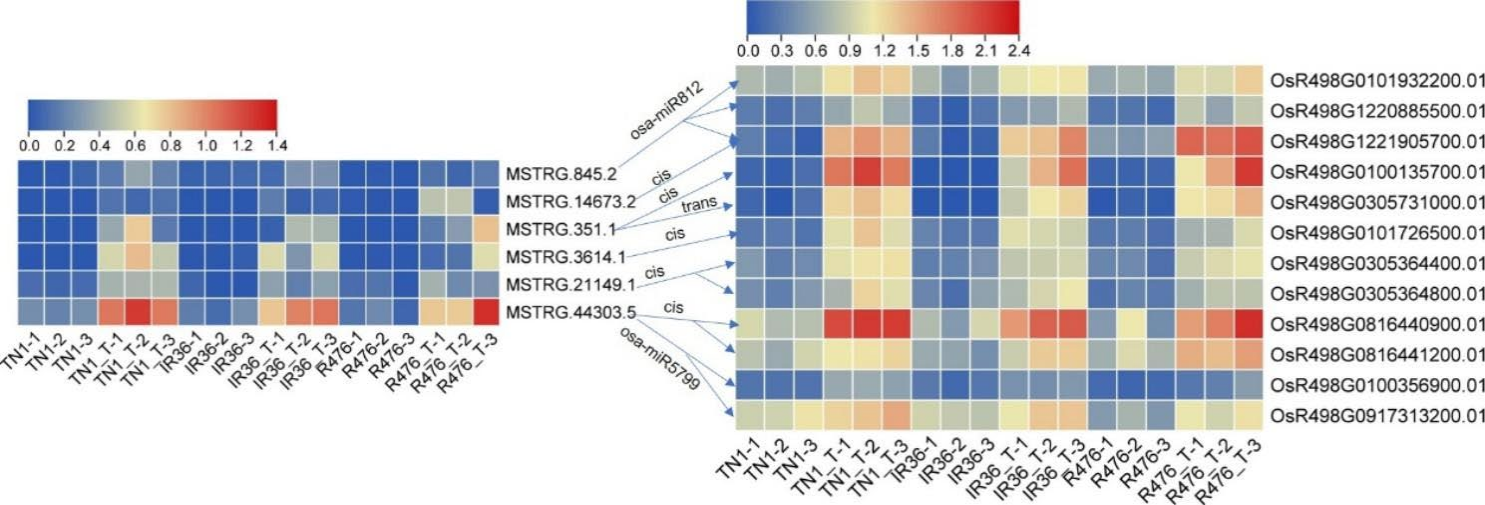
The expression of common lncRNAs and their differentially expressed targets. log^FPKM^ was used for heatmap

### DELs between susceptible and resistant varieties before BPH feeding

**Fig. 6:**
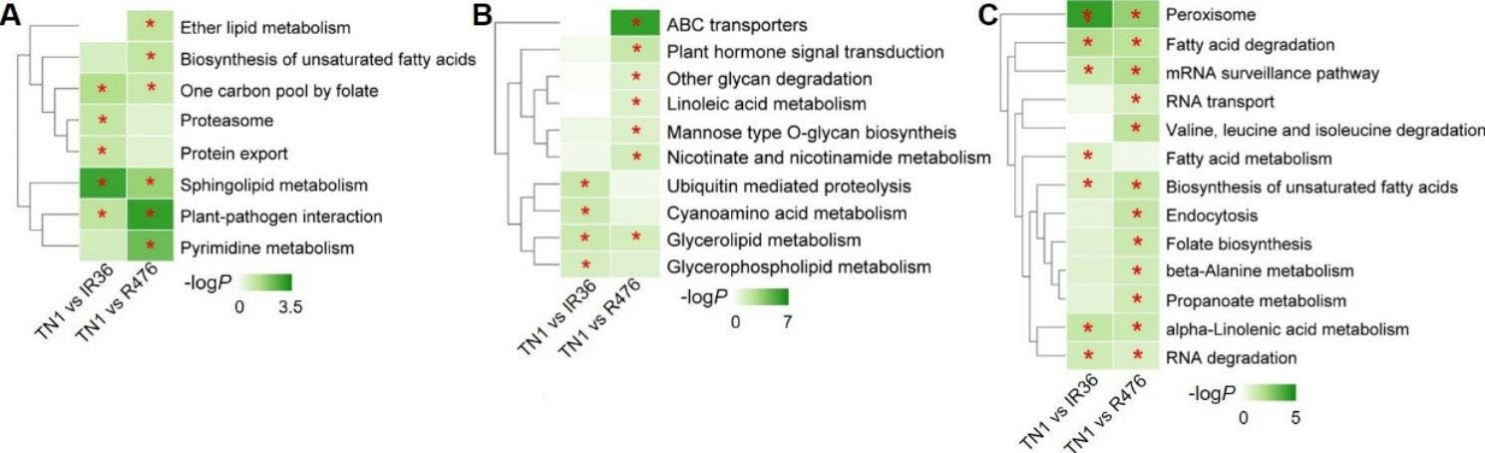
KEGG analysis of cis- **(a)**, trans- **(b)**, and target mimic-function **(c)** of DELs between susceptible (TN1) and resistant (IR36 and R476) varieties before BPH infestation. -log*P* was used for heatmap. * indicates *P* ≤ 0.05

It was noticed that during BPH infestation, the plant-pathogen interaction pathway directly related to defense was significantly enriched in the targets of DELs in the resistant variety IR36, while not in resistant variety R476 (Fig. 3b). Therefore, the GO and KEGG analysis of the putative targets of the DELs between susceptible and resistant varieties before BPH feeding were conducted and the significant items of KEGG pathways were shown in the heatmap (Fig. 6; Supplementary Table S12). Intriguingly, although the plant-pathogen interaction pathway was significantly enriched in both TN1 vs. IR36 and TN1 vs. R476 before BPH attacking, the DELs in TN1 vs. R476 could target more differentially expressed components of the plant-pathogen interaction pathway than those in TN1 vs. IR36 (Supplementary Table S12). In addition, some other pathways related to defense such as ABC transporters and glycan metabolism, were also found to be enriched specifically in the targets of DELs in TN1 vs. R476 before BPH attacking (Fig. 6b). What’s more, more DELs in TN1 vs. R476 were predicted to target more NBS-LRR and RLK genes than those in TN1 vs. IR36 (Supplementary Table S13, S14). The results indicated that these DELs targeting defense related pathways or resistant proteins are important in the R476 and IR36 prior to BPH feeding. By the regulation of the lncRNAs, R476 were in a priming state, probably contributing to the more BPH resistant phenotype of R476 than IR36.

In particular, MSTRG.12146.1, exclusively expressed in R476 (Supplementary Table S2), were predicted to target 12 protein coding genes by cis- or trans- activity (Supplementary Table S14). Among these targets, 9 genes encode resistant proteins containing NB-ARC domain, 6 of which were differentially expressed between TN1 and R476 before BPH feeding (Supplementary Table S14; Supplementary Fig. S5). Furthermore, all the 6 NBS-LRR genes were located on Chromosome 11, with which MSTRG.12146.1 showed complementary sequence on the region of upstream and the 1st exon. We speculated that MSTRG.12146.1 could target several genes encoding NBS-LRR protein to fine-tune the resistance to BPH in resistant rice R476.

### Analysis of lncRNAs extensively participating in BPH defense in either susceptible or resistant varieties by WGCNA

**Fig. 7:**
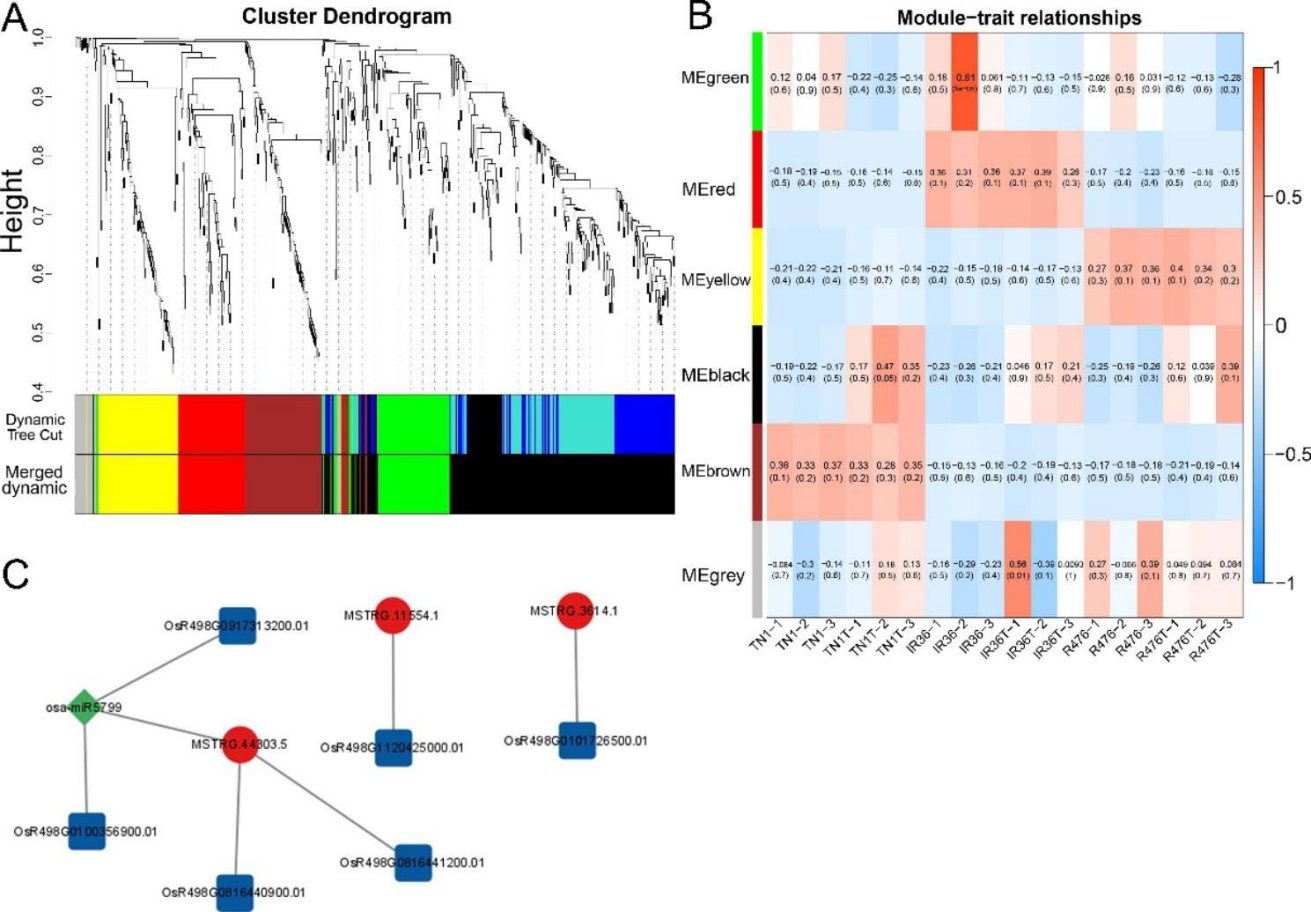
WGCNA of DELs and their putative target protein-coding genes involved in BPH response. **(a)** Hierarchical cluster tree indicating six modules identified by WGCNA. Each leaf in the tree is one gene and the major tree branches constitute six modules labeled by different colors. **(b)** Module-trait correlations and corresponding p-values. Each row corresponds to a module and each column corresponds to a sample. Each cell at the row-column intersection is color-coded by correlation according to the color legend. **(c)** The regulation network of lncRNAs and putative target protein-coding genes in “ME black” module constructed by cytoscape

To systematically explore the potential regulation functions of lncRNAs extensively associated with defending BPH in either susceptible or resistant variety. WGCNA was performed to analyze all the 631 DELs and their 4547 putative targets (Supplementary Table S15). A total of 6 distinct modules were obtained by analyzing the module-trait correlations (Fig. 7a). Of particular interest, of the 6 modules, the “ME black” module displayed an upregulation trend in each variety after BPH attacking, and was in line with the BPH attacking in the 3 varieties. Subsequently, the ME black module was suspected to contain key lncRNAs and mRNAs which extensively participate in BPH defense regardless of varieties and was selected to be investigated further (Fig. 7b). The “ME black” module comprised 3 DELs (MSTRG.3614.1, MSTRG.44303.5 and MSTRG.11554.1) and 171 protein-coding genes, in which 6 were predicted as the targets of these 3 DELs (Supplementary Table S16). These 3 DELs from “ME black” module were selected as the most interested lncRNA extensively associated with defending BPH. Accordingly, the sub-network of these 3 key lncRNAs and their targets in the “ME black” module was construct (Fig. 7c).

## Discussion

Evidence is accumulating for a role of lncRNAs in the plant response to biotic stress [39, 41]. In rice, lncRNAs response to several pathogens have been identified, such as *Maganaporthe oryzae*, *Xanthomonas oryzae*, *Meloidogyne graminicola*, and rice black-streaked dwarf virus [39, 49, 59–61]. In plant-insect interactions, *Manduca sexta*-elicited and armyworm-elicited lncRNA expression have been studied in wild tobacco and rice, individually [41, 62]. These studies focused on the lncRNA expression pattern in one rice variety over time under biotic stress and predicted the lncRNA-targeted protein-coding genes by co-expression. However, it remains unknown whether if lncRNAs respond differentially to BPH feeding, in resistant and susceptible rice plants. This is the first report of the lncRNA response to BPH in resistant and susceptible rice. In this study, we compared the lncRNA expression between the resistant (IR36 and R476) and susceptible (TN1) rice plants before and after BPH attack. Furthermore, we identified the putative target protein-coding genes of lncRNAs by cis-, trans- and target mimic activity and constructed a network regulated by lncRNA extensively responding against BPH in resistant and susceptible rice. A total of 2283 lncRNAs were identified, among which, 84, 52 and 63 specific lncRNAs in TN1, IR36 and R476, respectively, were differentially regulated during BPH infestation (Supplementary Table S2). GO and KEGG analysis revealed that the predicted targets were enriched in various and quite different categories in different varieties (Fig. 3; Supplementary Table S12). Our results suggested that lncRNAs regulate the defense responses of rice to BPH by targeting protein coding genes engaged in different pathways. Our work not only provides a detailed snapshot of the lncRNA expression pattern in resistant and susceptible host rice after BPH attack, but also proposed the possible lncRNA-mRNA and lncRNA-miRNA-mRNA ceRNA networks against BPH. However, the function of lncRNAs in BPH infestation and the relationships of lncRNAs and their targets remain to be further confirmed.

### Roles of lncRNA-miRNA-mRNA ceRNA network in response to BPH feeding

**Fig. 8:**
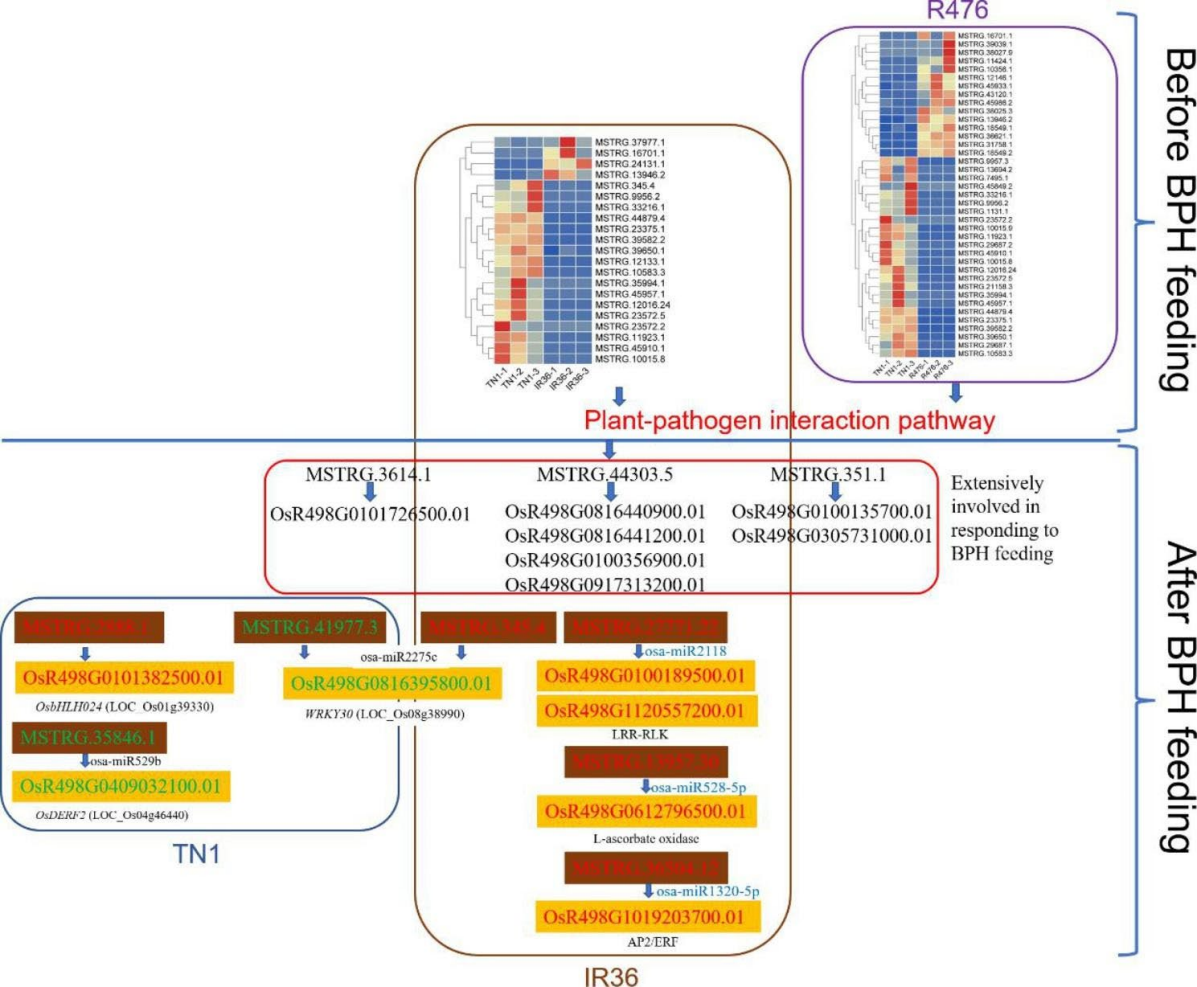
The diagram of molecular networks of key lncRNAs regulating BPH defense through well-studied stress-related genes and miRNAs before and after BPH feeding. Brown and yellow boxes indicate lncRNAs and mRNA, respectively. Red and green text represents upregulation and downregulation, respectively

LncRNAs can function as miRNA target to compete with mRNA for miRNA, forming a ceRNA network and thus regulating the expression of mRNA [48]. In our study, beyond the cis- and trans-function, the target mimic activity of lncRNA was analyzed. miR2118 super-family has been well characterized to target *NBS-LRR* genes in tomato (*Solanum lycopersicum*) and *Medicago truncatula* in the regulation of immune response [63, 64]. In the present study, MSTRG.27771.22, which was induced by BPH feeding specifically in the resistant variety IR36, was predicted as the target for osa-miR2118 family (Supplementary Table S9; Fig. 8). Instead of NBS-LRR genes, several other targets such as protein kinase (OsR498G0100189500.01) and LRR-RLK (OsR498G1120557200.01) were upregulated. This indicated that miR2118 probably fine-tune BPH defense by targeting other defense-related genes other than *NBS-LRRs* through the target mimic regulation of MSTRG.27771.22. Osa-miR528/L-Ascorbate Oxidase module regulated by OsSPL9 enhances anti-viral defense in rice [53, 65]. In the present study, both MSTRG.13957.30, a putative target lncRNA for osa-miR528-5p, and the target mRNA for osa-miR528, OsR498G0612796500.01 (L-ascorbate oxidase), was upregulated in IR36 during BPH infestation (Supplementary Table S5, S9). A recent report showed that Osa-miR1320 overexpressing lines displayed an enhanced resistant phenotype to rice blast [66]. Osa-miR1320 targets the ERF transcription factor OsERF096 to regulate cold tolerance via JA-mediated signaling [67]. Additionally, osa-miR395, osa-miR812, and osa-miR1320 have also been found to be differentially regulated in rice under BPH feeding [18]. In our study, it was also found that MSTRG.897.1, MSTRG.36504.12, and MSTRG.8977.8 could compete with the targeting protein-coding genes for osa-miR395 family, osa-miR1320-5p, and miR812 family, respectively (Supplementary Table S5; Fig. 8). Taken together, we speculated that these lncRNA-miRNA-mRNA ceRNA networks acted a vital role in defending against BPH attacking.

LncRNAs targeting resistance genes were involved in response to BPH feeding.

Previous studies have proposed that plants’ defense to insects shares many similarities with their defense to pathogens [2, 51, 52]. Kourelis and van der Hoorn (2018) reviewed that, of the cloned resistance genes, 61% encode NBS-LRRs [54]. The other main class of cloned resistance genes (19%) encode RLPs/RLKs. In our study, *NBS-LRR* genes targeted by DELs were found to be upregulated after BPH feeding in TN1 and R476 (Supplementary Table S8, S10; Fig. 4a, c). It was also found that several RLPs/RLKs genes targeted by 5, 5, and 7 specific DELs in TN1, IR36 and R476 were differentially expressed after BPH feeding (Supplementary Table S8, S9, S10; Fig. 4a-c). Overall, based on these results, we supposed that lncRNAs targeting resistance genes were involved in response to BPH feeding.

### LncRNAs targeting transcription factors were involved in response to BPH feeding

Several genes encoding transcription factors have been found to regulate the BPH defense in rice. *Bph29*, containing a B3 DNA-binding domain, activates the SA signaling and suppresses the JA/ethylene-dependent pathway in response to BPH infestation [68]. Overexpression of *WRKY89* enhanced resistance to white-backed planthopper [69]. MYB30 upregulates the expression of *OsPALs* to enhance the biosynthesis and accumulation of SA and lignin [7]. BPH14 interacts with transcription factors WRKY46 and WRKY72 to activate the expression of the receptor-like cytoplasmic kinase and the callose synthase genes [15]. In our study, a few lncRNAs targeting transcription factors such as MYBs and WRKYs, were induced by BPH infestation (Supplementary Table S8, S9, S10; Fig. 4a-c). OsWRKY30 (OsR498G0816395800.01/LOC_Os08g38990) increases the endogenous JA accumulation, *PATHOGENESIS-RELATED* (*PR*) gene expression and resistance to fungal pathogens and *Xanthomonas oryzae* in rice [70, 71]. In the present study, OsWRKY30, which was downregulated among all the 3 varieties upon BPH feeding, was putatively targeted by miR2275c. MSTRG.41977.3 downregulated in TN1 and MSTRG.345.4 upregulated in IR36 could function as target mimic for miR2275c (Supplementary Table S8, S9; Figs. 4a and b and 8). OsbHLH024 (OsR498G0101382500.01/LOC_Os01g39330) was implied to be a negative regulator of salt stress revealed by enhanced resistance of *osbhlh024* knockout plants [72]. The heat shock factor (HSF) gene, *OsHsfC2a* (OsR498G0203284300.01/LOC_Os02g13800), has been reported to have increased expression under a few abiotic stresses, i.e. heat, cold, oxidative stress, submergence [73, 74] as well as drought and desiccation [75, 76]. This transcription factor also showed response to brassinosteroids (BR), SA and abscisic acid (ABA) [75, 76]. Promoter analysis of *OsHsfC2a* revealed presence of light, ABA and methyl-jasmonate (MeJA) responsive cis-elements [77]. Besides, *OsDERF2* (OsR498G0409032100.01/LOC_Os04g46440) knock-down lines enhanced tolerance to drought stress at seedling stage and were much more sensitive to ABA [78]. At the present study, MSTRG.2888.1 and MSTRG.16452.2 were predicted to target *OsbHLH024* and *OsHsfC2a* in a cis-way in TN1, while MSTRG.35846.1 putatively competitived with *OsDERF2* for osa-miR529b (Supplementary Table S8; Figs. 4a and 8). Based on all above evidences and results, it is proposed that lncRNAs may play important roles in BPH resistance by involving in targeting transcription factors.

A priming defense state in R476 regulated by lncRNA might contribute to the defense against BPH attacking.

R476 displayed a more resistance phenotype than IR36 revealed by the ecological fitness of BPH reared on IR36 and R476 (Fig. 1). However, during BPH infestation, the plant-pathogen interaction pathway was significantly enriched targeted by trans-function of DELs in the resistant variety IR36, while not in resistant variety R476 (Fig. 3). Intriguingly, before BPH attacking, although the plant-pathogen interaction pathway was significantly enriched in both IR36 and R476, more DELs in R476 could target more components of the plant-pathogen interaction pathway than those in IR36 (Figs. 6 and 8; Supplementary Table S12). In addition, some other pathways related to defense such as ABC transporters and glycan metabolism, were also found to be enriched specifically in the targets of DELs in R476 before BPH attacking (Fig. 6; Supplementary Table S12). The results indicated that these DELs targeting defense related pathways or resistant proteins might be important in the IR36 and R476 prior to BPH feeding. By the regulation of the lncRNAs, R476 were in a priming state, probably contributing to the more BPH resistant phenotype of R476 than IR36.

### Roles of key lncRNAs extensively involved in BPH defense

After BPH infestation, 7 lncRNAs were commonly differentially expressed among the three varieties (Supplementary Table S11; Fig. 5). Of these DELs, MSTRG.3614.1 and MSTRG.44303.5 were also found in “ME black” module which were extensively involved in BPH response in both susceptible and resistant varieties (Fig. 7; Supplementary Table S16). Particularly, four targets of MSTRG.44303.5 were also included in the “ME black” module. Of these targets, *LOX8* (OsR498G0816440900.01) could catalyze the formation of oxidized fatty acids, supplying vital substrates for JA [58]. The other DE targets of MSTRG.44303.5 were involved in carbohydrate transport and metabolism (Supplementary Table S11). Additionally, MSTRG.44303.5 putatively act as the target mimic for osa-miR5799, and its putative target PR protein 1 (OsR498G0713441800.01), a class of proteins activated in response to different biotic and abiotic threats, was upregulated upon BPH infestation. On the other hand, lncRNA *ELENA1* directly interacts with Mediator subunit 19a (MED19a) and affects enrichment of MED19a on the *PR1* promoter to enhance expression of innate immune response genes in Arabidopsis [79, 80]. In our study, lncRNA MSTRG.44303.5 may regulate *PR1* in the same way as *ELENA1*. These results implied that MSTRG.44303.5 could be a conserved lncRNA extensively contributing to basal defense against BPH feeding in multiple ways.

Plants may use flavonoids to deter the feeding, development, and oviposition of herbivores [81]. Several flavonoids respond to insects, for example, vitexin inhibits *Spodoptera litura* larval growth [82] and schaftoside inhibits BPH growth [8, 83]. The OsmiR396-OsGRF8-OsF3H (flavanone 3-hydroxylase) module mediates BPH resistance through flavonoid biosynthesis [8]. OsF3H was further demonstrated to positively regulate the resistance to BPH [9]. In the current study, it was found that the expression of leucocyanidin oxygenase in flavonoid biosynthesis pathway (OsR498G0305731000.01/LOC_Os03g18030) trans-targeted by MSTRG.351.1 significantly increased after BPH infestation among 3 varieties (Supplementary Table S11).

## Conclusion

In summary, a total of 2283 lncRNAs were identified, of which 649 lncRNAs were differentially expressed. During BPH infestation, 84, 52 and 63 specific DELs were found in TN1, IR36 and R476, respectively. The lncRNAs targeting the well-studied stress-related miRNAs, resistance genes (*NBS-LRRs* and *RLKs*), transcription factors in each variety were identified. The significantly enriched plant-pathogen interaction pathway in the resistant rice before BPH feeding raised the possibility of a priming state by the regulation of lncRNAs. Additionally, WGCNA revealed the lncRNAs extensively involved in response to BPH feeding and the possible regulation networks of the key lncRNAs were constructed. These findings will provide further understanding of the regulatory roles of lncRNAs in BPH defense, and lay a foundation for functional research on the candidate lncRNAs.

## Materials and methods

### Plant and insect materials

Three indica rice varieties were used in this study. TN1 was susceptible rice, while IR36 (harboring *Bph2*) and R476 (harboring *Bph14* and *Xa21*) were BPH-resistant varieties provided by the Zhejiang Academy of Agricultural Sciences (Hangzhou, China) and Sun Yat-sen University (Guangzhou, China). The rice seeds were sown in a soil-filled tub (1 m × 0.5 m × 0.15 m) for 7 d. 7-day-old rice seedling was transplanted into a plastic pot (20 cm × 10 cm). 45-day-old rice plants at the tillering stage were used for the subsequent experiments. The BPH population were reared on the susceptible TN1 rice variety for more than 10 years under controlled environmental conditions (26 ± 1℃), 16:8 h light:dark cycle, 70–90% relative humidity).

### Measurement of the ecological fitness of BPH reared on TN1, IR36 and R476

The BPH ecological fitness measurement was conducted based on the method as previously described [84]. The TN1, IR36 and R476 rice plants were separately placed into a test tube (2.5 cm ×15 cm) filled with 15 mL rice nutrient solution and sealed with cotton. The rice plants in the test tube were infested with twenty newly hatched BPH nymphs (< 12 h). At least twenty replicates were used for each rice group. The observation of nymph development was performed daily until adulthood, and rice plants of three varieties were replaced frequently and simultaneously to ensure the supply of fresh food. The survival number of nymphs and the days when each nymph becomes an adult were recorded. All newly emerged adults (< 12 h) in each treatment were weighed using an electronic balance (0.01 mg) (Sartorius, Gottingen, NI, GER). A pair of newly emerged BPH from each rice variety was placed into a test tube prepared as described above. Twenty replicates were conducted for each treatment group. The survival days of female adult in each replicate was recorded. The newly hatched BPH nymphs (< 12 h) from each replicate were recorded in detail and removed until no nymphs hatched for three consecutive days. The number of unhatched eggs was counted by dissecting rice plants. All experiments were carried out under controlled environmental conditions as described above.

### Sample collection for lncRNA transcriptome analysis

The rice samples were collected through an improved method based on the protocol described by Liu et al., (2020) [84]. Experimental sachets (5 cm in length and 3 cm in width) were prepared using parafilm membranes (BEMIS, Neenah, WI, USA). Five were introduced into each bag, and the bag introducing five newly emerged BPH adults (as BPH treatment) and empty bag (as CK) was fixed to the stem of each rice varieties, individually 2 cm above ground level so that the whole bag was fully stretched, and the pocket opening was tightly closed. After 24 h, the treatment in which all BPH in the experimental sachet survived was used as an effective repeat. The outer leaf sheath of the stem of rice was precisely cut off. Fifteen independent treatments were performed for each rice variety, and then were pooled into triplicates for lncRNA transcriptome sequencing. Samples without BPH feeding were referred as TN1, IR36 and R476, while samples with BPH feeding were referred as TN1_T, IR36_T and R476_T.

### RNA extraction, library construction and sequencing

Total RNAs from samples were extracted by using TRIzol reagent (Invitrogen, USA), according to the instructions. The integrate and qualified RNAs were firstly removed ribosomal RNA, and then were fragmented using divalent cations under elevated temperature. The fragmented mRNAs were then used for constructing strand specific cDNA library followed by the standard Illumina protocols and then sequenced on an Illumina HiSeq™ 4000 sequencing platform (Illumina, San Diego, CA, USA). Constructions of libraries and RNA sequencing were performed by Biomarker Technologies Co., Ltd. (Qingdao, Shandong, China).

Identification of mRNAs and lncRNAs.

The clean reads were obtained by filtering out raw reads containing adapter, ploy-N and low-quality reads using Sickle and SeqPrep software, and then were aligned to the R498 reference genome (Indica cv.) (http://www.mbkbase.org/R498/) using HISAT2 software [85]. The mapped reads were further assembled by StringTie [86]. Next, the assembled transcripts were annotated by using the gffcompare program to identify protein-coding mRNAs. The remained unknown transcripts with lengths more than 200 nt and more than two exons were selected as lncRNA candidates [87]. The final lncRNAs were acquired by further filtering the putative protein-coding RNAs using four computational approaches including CPC2 (CPC score > 0) [88], CNCI (CNCI score > 0) [89], Pfam [90] and CPAT [91].

### Differential expression analysis of mRNAs and lncRNAs

The expression of both lncRNAs and mRNAs in each sample was quantified using fragments per kilobase of exon per million fragments mapped (FPKM) calculated by StringTie software. The transcripts with FPKM less than 0.1 were filtered out as low expression. Pairwise differential expression analyses between BPH infested and mock plants (including TN1 vs. TN1_T, IR36 vs. IR36_T, and R476 vs. R476_T), between susceptible and resistant variety before BPH-infested (including TN1 vs. IR36 and TN1 vs. R476), and between susceptible and resistant variety after BPH-infested (including TN1_T vs. IR36_T and TN1_T vs. R476_T) were performed by using the DESeq2 package with read counts in R [92]. The DEGs or lncRNAs (DELs) were filtered by the threshold of a statistical significance *P* < 0.05 and an absolute value of log2 (Fold Change) > 1.

### Target gene prediction and ceRNA identification of lncRNAs

Potential target genes of the lncRNAs were predicted based on their regulatory patterns including cis and trans-acting groups. The potential cis-target genes were identified by searching the protein-coding genes colocalized within the 10 kb upstream or downstream of individual lncRNAs by using Perl script [93]. Furthermore, the trans-targets of lncRNAs were predicted based on the complementarity between lncRNA and protein-coding genes by LncTar [94]. Moreover, lncRNAs function as miRNA targets to form ceRNA (competing endogenous RNAs) networks and thus indirectly regulate the expression of target transcripts of the same miRNAs [93]. Thus, the potential target mimic function of the lncRNAs was evaluated by psRNAtarget (https://www.zhaolab.org/psRNATarget/) [95]. All sequences of the interested lncRNAs were subjected to identify miRNA (expectation value > 3.0). After retrieving their sequences from miRbase (version 21), these miRNA sequences were entered in psRNAtarget to find putative mRNA targets (expectation value > 3.0) [96].

### Function classification for the target mRNAs of interested lncRNAs

GO enrichment of the targets of the interested lncRNAs were implemented using Blast2GO (http://www.blast2go.org/) [97], and GO terms with p-value < 0.05 were considered significantly enriched. The targets of the interested lncRNAs were annotated by KEGG Automatic Annotation Server based on the KEGG database (http://www.genome.jp/kegg/)[42–44]. The p-value < 0.05 was required for differences to be considered statistically significant.

WGCNA of DELs and their potential target protein-coding genes.

To further explore the functions of lncRNAs related to BPH resistance, WGCNA was conducted using the R package [98]. An unsigned co-expression relationship was built based on the adjacency matrix between DELs and their putative cis-, trans-, and target mimic-target protein-coding genes. Genes with low FPKM values (average FPKM < 0.1) were excluded. A threshold of 4 was interpreted as a soft power for the correlation matrix. The minimum module size and the minimum height of the merging modules were set to 30 and 0.5, respectively. Genes were clustered hierarchically according to the topological-overlap matrix measure. Highly similar modules were subsequently identified by clustering and then merged into new modules based on eigengenes. The correlation of each module was also analyzed and visualized by a heatmap. Finally, the network of the interested module was visualized by Cytoscape software.

### Validation of DEGs and DELs by RT-qPCR

Six lncRNAs and six protein-coding genes were randomly selected for RT-qPCR validation of transcriptome (Supplementary Table S17). The qualified RNA (1 µg) was used for reverse transcription to synthesize cDNA using the PrimeScript™ RT reagent kit (Takara Bio, Inc., Otsu, Shiga, Japan). Gene-specific primers for selected genes were designed using Primer Blast, and the *actin* gene was used as an internal control. qPCR was performed on a Light Cycler 480 System (Roche Diagnostics, Basel, Switzerland) and the ChamQ Universal SYBR qPCR Kit (Vazyme Biotech Co., Ltd, Nanjing, China) according to the instructions provided by the manufacturer. A 10 µL reaction mixture was constructed, with each reaction mixture containing 1 µL cDNA, 0.3 µL each of 10 µmol·L^− 1^ primers, and 5 µL Universal SYBR master mix (Vazyme Biotech Co., Ltd, Nanjing, China). The PCR amplification conditions were as follows: 5 min at 95 °C, followed by 45 cycles of 95 °C for 10 s, 20 s at 60 °C, and 20 s at 72 °C. Three biological replicates and three reactions were performed for each sample. Changes in gene expression were evaluated by the 2^−∆∆Ct^ method [99]. The primers used in the qPCR experiments are listed in Supplementary Table S17.

## Electronic supplementary material

Below is the link to the electronic supplementary material.

Supplementary Material 1

Supplementary Material 2

## Data Availability

The transcriptome data generated in this study are deposited in the NCBI SRA database under BioProject PRJNA894795. The names of the repository/repositories and accession number(s) can be found in the article/**Supplementary Material**, further inquiries can be directed to the corresponding author/s.

## Abbreviations

LncRNAs: Long non-coding RNAs
BPH: Brown planthopper
TN1: Taichung Native 1
DELs: Differentially expressed lncRNAs
WGCNA: Weighted Gene Co-expression Network Analysis
SA: Salicylic acid
JA: Jasmonic acid
lincRNA: intergenic lncRNA
incRNA: intronic lncRNA
NAT: Natural antisense lncRNA
GO: Gene Ontology
DEGs: Differentially expressed genes
KEGG: Kyoto Encyclopedia of Genes and Genomes
RT-qPCR: Quantitative real-time polymerase chain reaction
ceRNA: Competing endogenous RNA
BR: Brassinosteroids
ABA: Abscisic acid
MeJA: Methyl-jasmonate
FPKM: Fragments per kilobase of exon per million fragments mapped
