# Supplementary material for: Comprehensive transcriptomic analysis of three varieties with different brown planthopper-resistance identifies leaf sheath lncRNAs in rice
Source: BMC Plant Biol. 2023 Jul 22;23:367. doi: 10.1186/s12870-023-04374-w (PMC10362764; doi:10.1186/s12870-023-04374-w)
Supplement: Supplementary file 1 — Supplementary Material 1 [file 12870_2023_4374_MOESM1_ESM.docx]

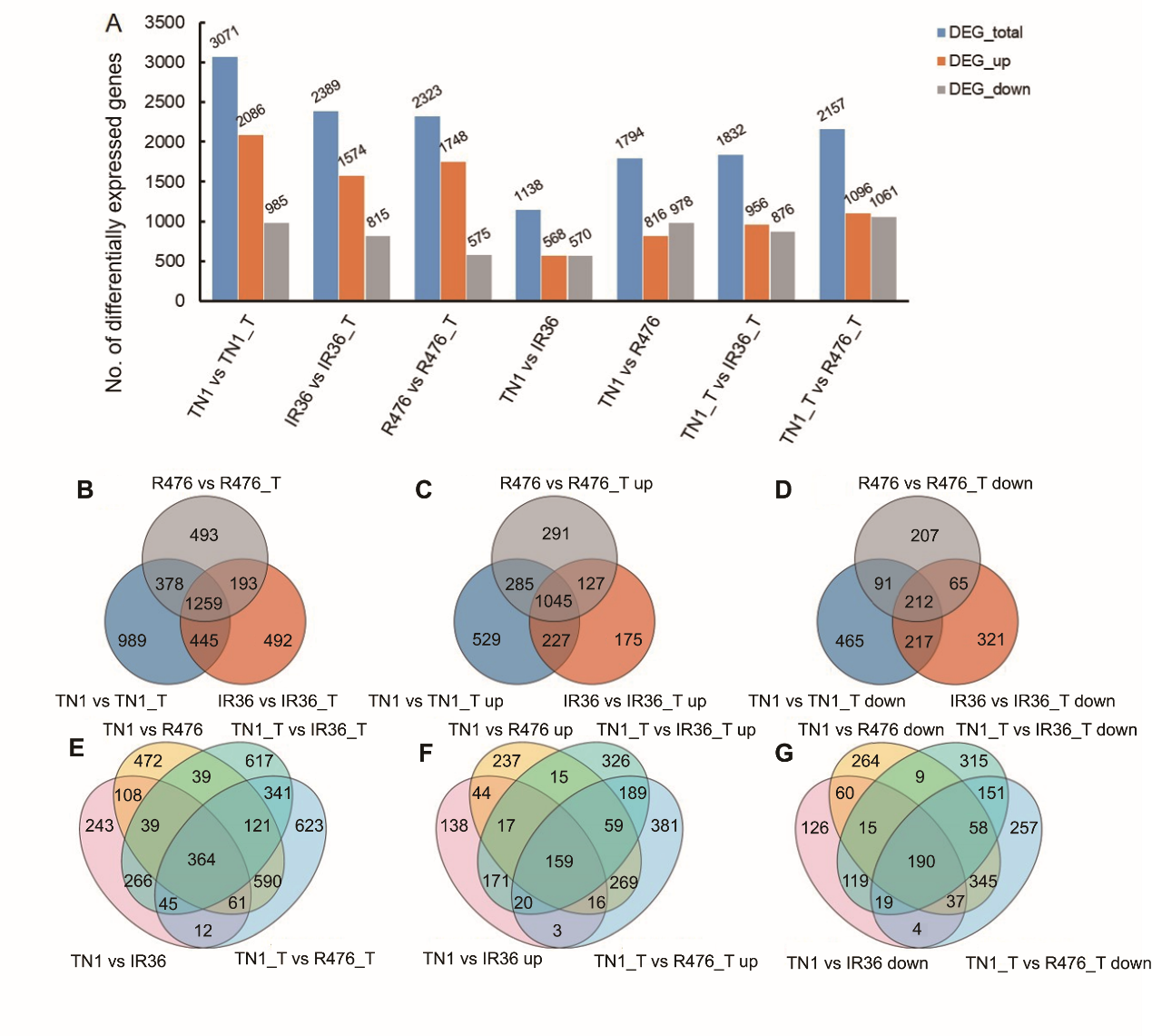


**Fig.** **S1** Differentially expressed genes (DEGs) in the comparisons. **(a)** Number of genes up- or downregulated in all comparisons (*P* ≤ 0.05, |log_2_ Fold Change| ≥ 1). **(b-d)** Venn diagrams of the total **(b)**, upregulated **(c)**, and downregulated **(d)** DEGs after BPH feeding in each variety (the comparisons of TN1 vs TN1_T, IR36 vs IR36_T, and R476 vs R476_T). **(e-g)** Venn diagrams of the total **(e)**, upregulated **(f)**, and downregulated **(g)** DEGs between susceptible and resistant variety before and after BPH feeding (the comparisons of TN1 vs IR36, TN1 vs R476, TN1_T vs IR36_T, and TN1_T vs R476_T).

**
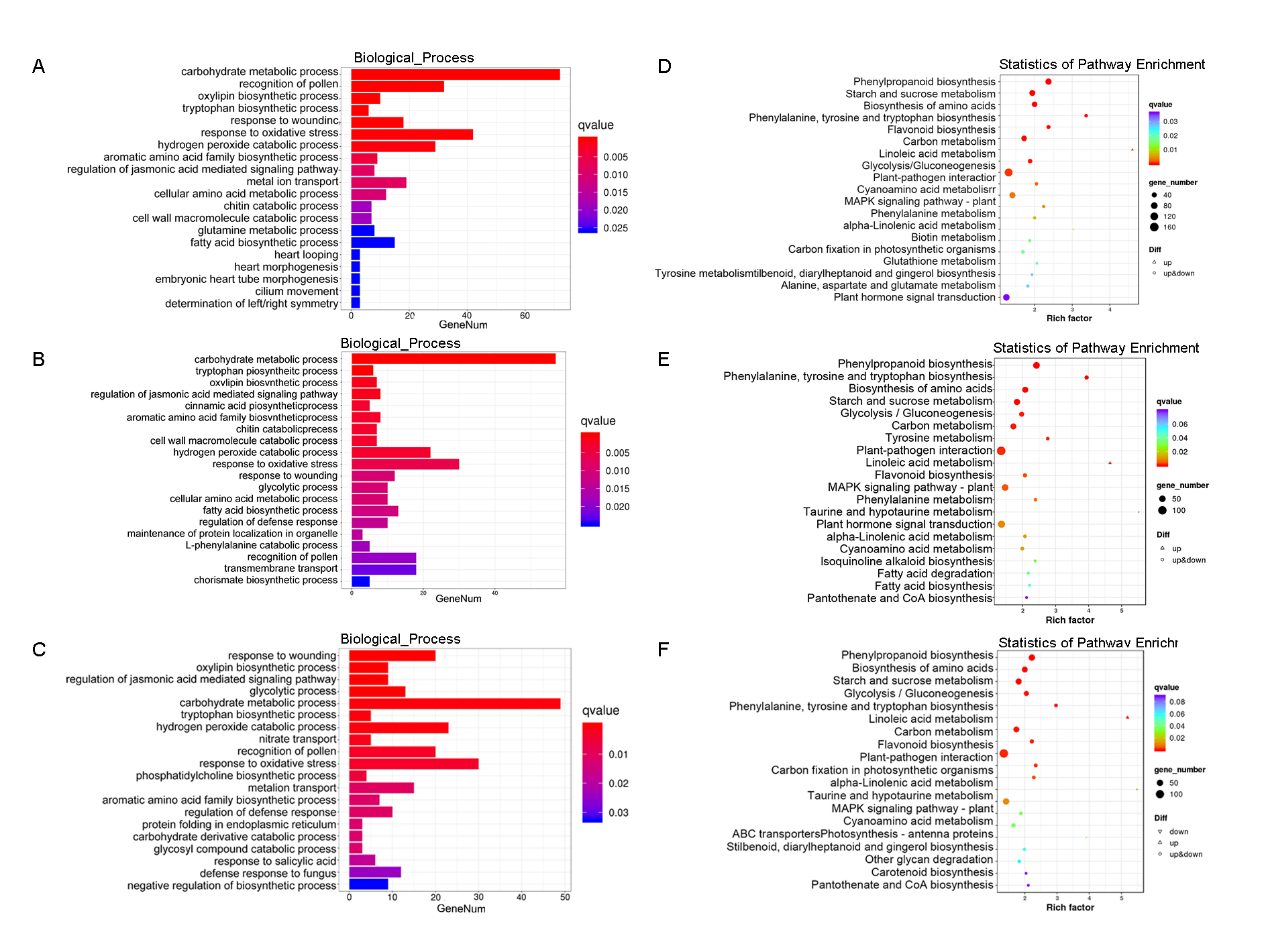
**

**Fig. S2** Gene Ontology (GO) and KEGG analysis of DEGs after BPH infestation in each variety including TN1, IR36 and R476. **(a-c)** Top 20 GO terms in biological processes of DEGs in TN1 **(a)**, IR36 **(b)**, and R476 **(c)** after BPH infestation. **(d-f)** Top 20 KEGG pathways of DEGs in TN1 **(d)**, IR36 **(e)**, and R476 **(f)** after BPH infestation.


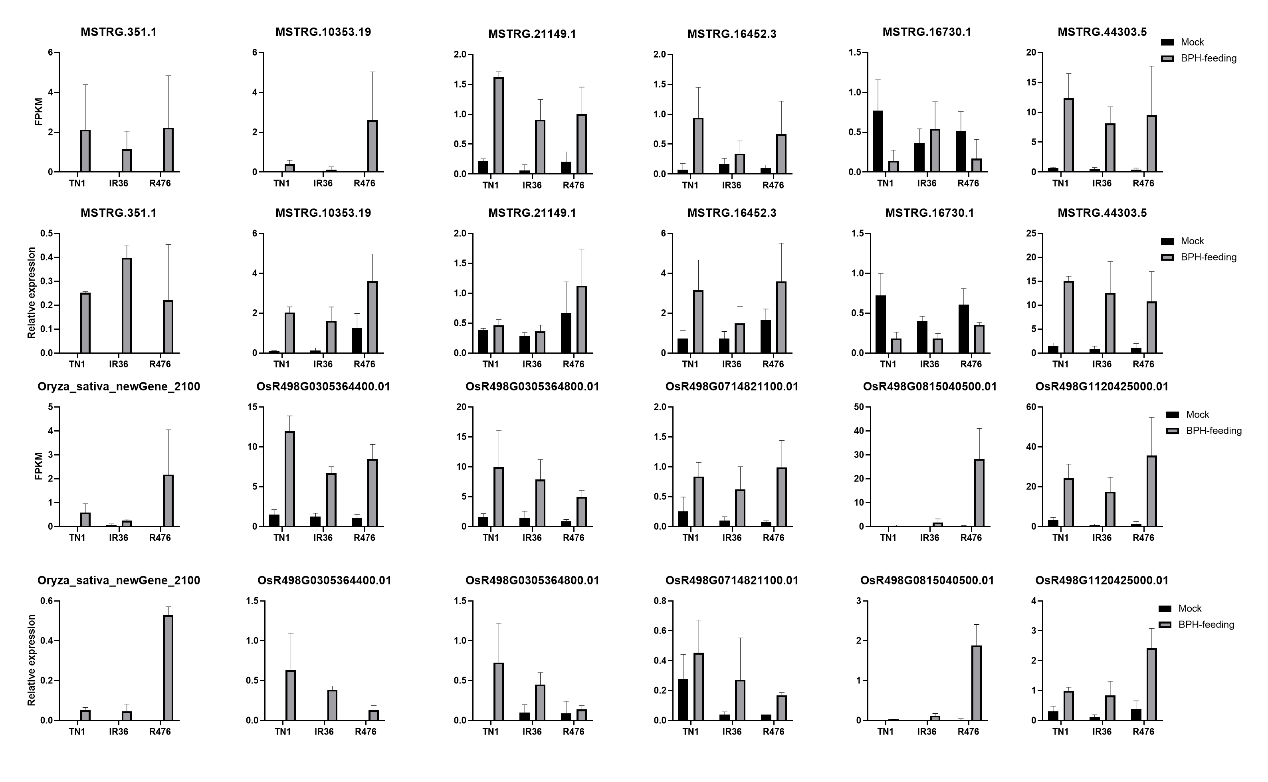
 **Fig. S3.** Comparison of the transcriptome analysis and qRT-qPCR validation. The values are presented as the mean ± SD (n = 3).


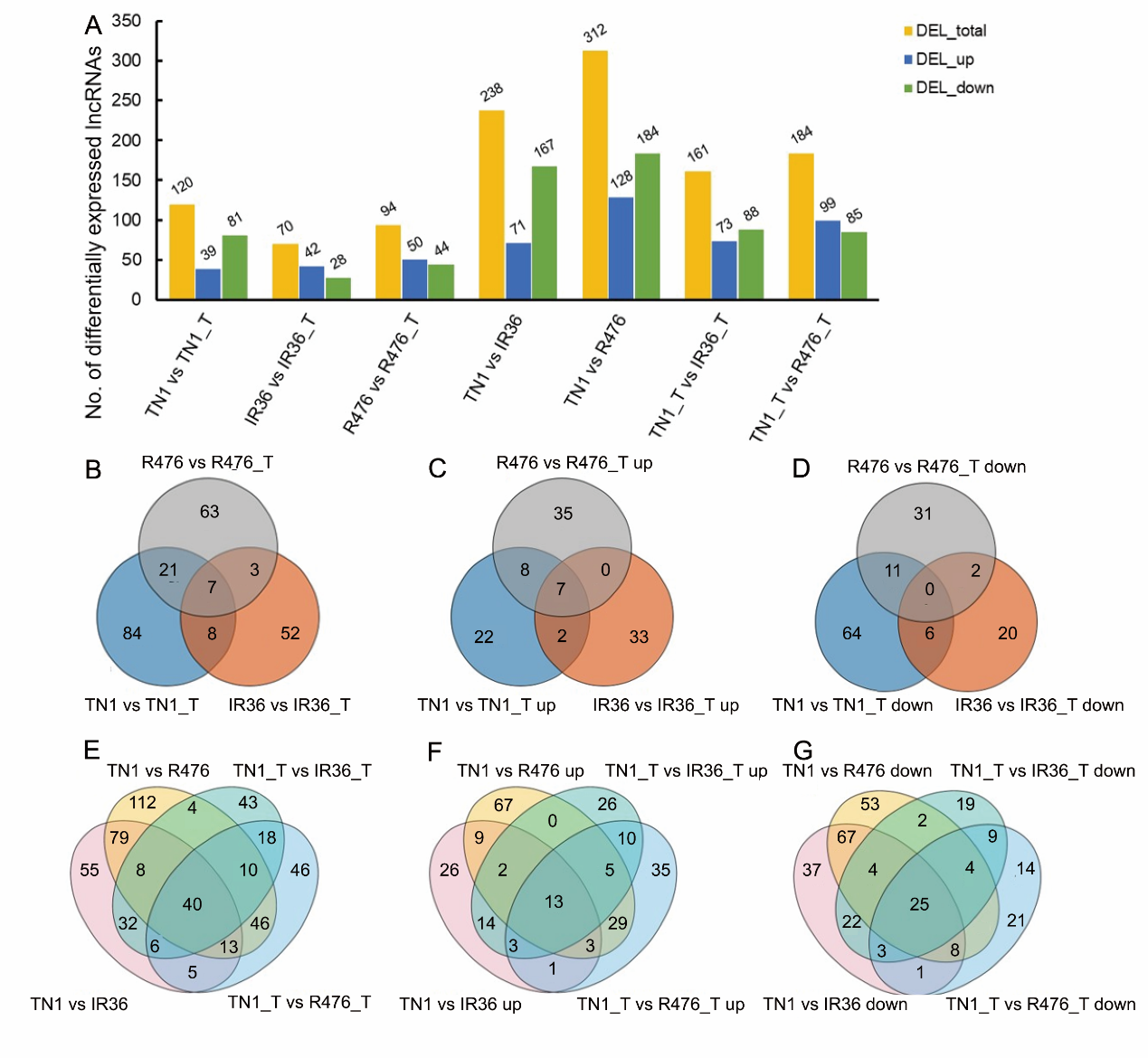


**Fig. S4** Differentially expressed lncRNAs (DELs) in all the comparisons. **(a)** Number of lncRNAs up- or downregulated in all comparisons (*P* ≤ 0.05, |log_2_ Fold Change| ≥ 1). **(b-d)** Venn diagrams of the total **(b)**, upregulated **(c)**, and downregulated **(d)** DELs after BPH feeding in each variety (the comparisons of TN1 vs TN1_T, IR36 vs IR36_T, and R476 vs R476_T). **(e-g)** Venn diagrams of the total **(e)**, upregulated **(f)**, and downregulated **(g)** DELs between susceptible and resistant variety before and after BPH feeding (the comparisons of TN1 vs IR36, TN1 vs R476, TN1_T vs IR36_T, and TN1_T vs R476_T).


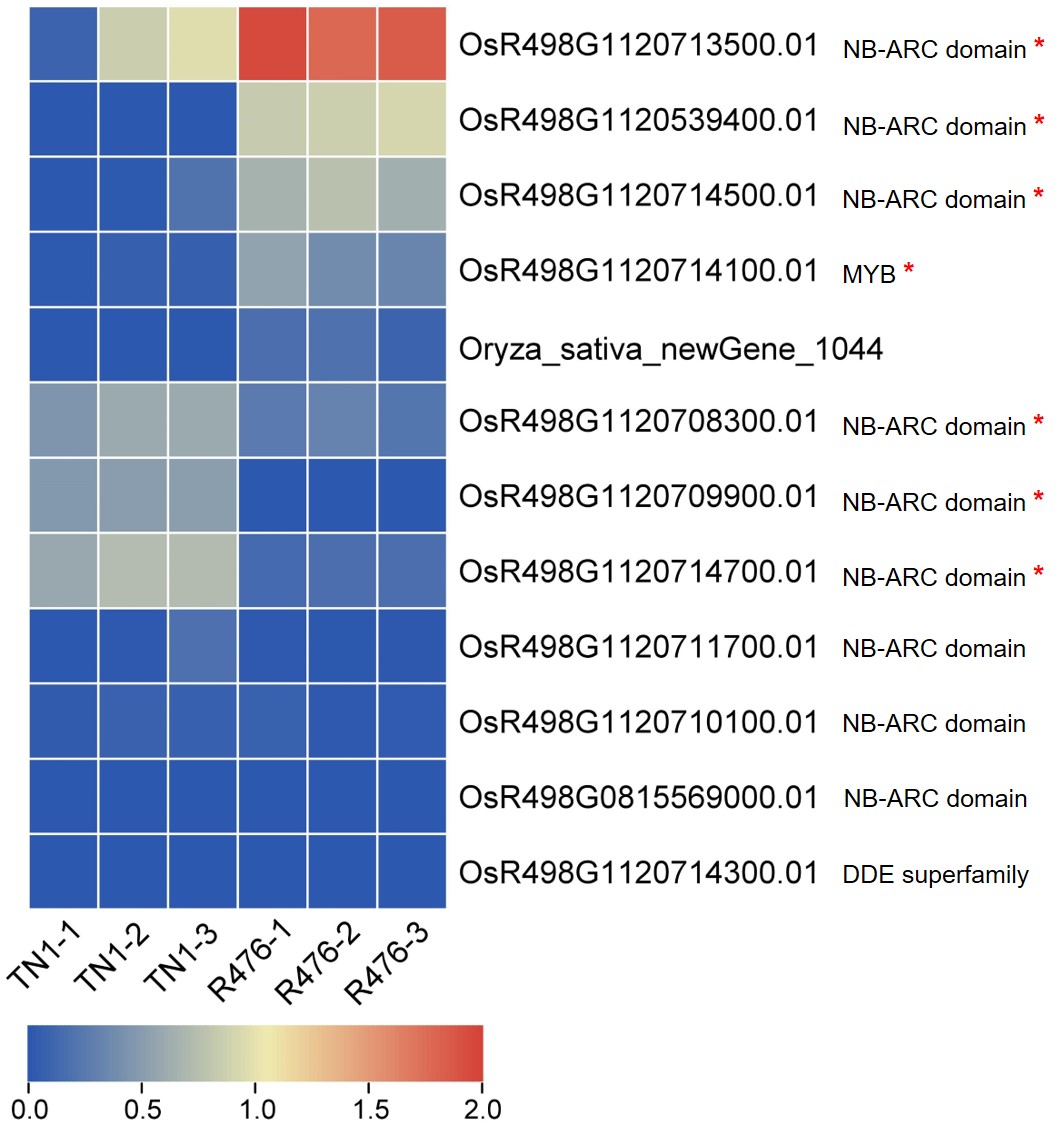


**Fig. S5** The expression of the putative targets of MSTRG.12146.1. log^FPKM^ was used for heatmap. * indicates differentially expressed at *P* ≤ 0.05
